# Ewing’s sarcoma with an uncommon clinical course: A case report

**DOI:** 10.3892/ol.2013.1320

**Published:** 2013-04-26

**Authors:** RUI NIIMI, AKIHIKO MATSUMINE, TOMIKI NAKAMURA, RYO MORIMOTO, TETSUYA MURATA, TAKASHI SUZUKI, YASUAKI NAKASHIMA, TAKAYUKI NOJIMA, ATSUMASA UCHIDA, AKIHIRO SUDO

**Affiliations:** 1 Department of Orthopaedic Surgery, Mie University Graduate School of Medicine, Tsu City, Mie 514-8507;; 2 Department of Pathology JA Suzuka General Hospital, Suzuka, Mie 513-8630;; 3 School of Pharmacy, Nihon University, Chiba 274-8555;; 4 Laboratory of Anatomic Pathology, Kyoto University Hospital, Sakyo-ku, Kyoto 606-8507;; 5 Department of Pathology and Laboratory Medicine, Kanazawa Medical University, Kahoku-gun, Ishikawa 920-0293, Japan

**Keywords:** Ewing’s sarcoma, recurrence, unusual clinical course

## Abstract

Here, a case of Ewing’s sarcoma family of tumors (ESFT) of the femur with an unusual clinical course is reported. At 20 years of age, the patient had undergone curettage of a bone tumor of the right femur which was diagnosed as ESFT. One cycle of chemotherapy with vincristine and cyclophosphamide and radiotherapy for a total dose of 40 Gy was administered. The patient did not develop any recurrence or metastases for the following 18 years, in spite of the inadequacy of the initial treatment. At 38 years of age, he was referred to our institution with right thigh pain that had persisted for several months. Radiographs and magnetic resonance imaging findings showed a mass lesion in his proximal femur extending to the soft tissue. An open biopsy was performed and the lesion was diagnosed as recurrence of ESFT, although a molecular biological investigation did not reveal any expression of the characteristic fusion genes that have previously been reported. The patient received standard multimodal therapy employing standard combination chemo-therapy for ESFT and wide surgical excision. The patient has been disease-free for 9 years since the treatment. This patient may have a rare subtype of ESFT with an unknown chromosomal translocation and relatively non-aggressive biological behavior.

## Introduction

The Ewing’s sarcoma family of tumors (ESFT) are highly malignant, metastatic, primitive small round cell tumors of the bone and soft tissue that affect children and adolescents. ESFT comprises morphologically heterogeneous tumors that are characterized by non-random chromosomal translocations between the EWS gene on chromosome 22q12 and one of several members of the E-twenty six (ETS) family of transcription factors. In ∼85% of cases of ESFT, the FLI1 gene on chromosome 11 is the fusion partner of EWS (EWSFLI1) (1,2); in ∼10%, the EWS fusion partner is the ERG gene on chromosome 22 (EWS-ERG) (3–5). Other ESFT family members have been identified as fusion partners of EWS, but these cases are rare. In <1% of cases, variant translocations, namely t(7;22)(p22;q12), t(17;22)(q12;q12) and t(2;22) (q33;q12), involving fusion of the EWS gene with ETV1, E1AF (also known as ETV4) and FEV genes, respectively, have been described (6–9).

Dramatic improvements in the survival of ESFT have been achieved for children and adolescents due to the development of multidisciplinary treatments, including multiple drug chemotherapy, refined surgical techniques and appropriate radiation therapy. Between 1975 and 2002, the 5-year survival rate has increased from 59 to 76% for children younger than 15 years and from 20 to 49% for adolescents aged 15 to 19 years (10). However, the current studies show that 30–40% of non-metastatic ESFT patients will still develop recurrent disease (local and/or metastatic) in spite of multidisciplinary treatment (11). The known prognostic factors for ESFT are tumor size or volume, serum lactate dehydrogenase (LDH) levels, axial localization and age (>15 years). Under treatment, a poor histological response to preoperative chemotherapy and incomplete or no surgery for local therapy are further adverse prognostic factors (12).

Here, a case of ESFT of the femur with an atypical clinical course is reported. At the local hospital, the patient received inadequate initial treatment which consisted of tumor curettage, chemotherapy with insufficient dose intensity and low-dose radiation therapy. In spite of the inadequate initial treatment, the patient had been disease-free for the subsequent 18 years. The patient was examined due to thigh pain following local recurrence 18 years after initial treatment and received standard multimodal therapy employing combination chemotherapy and wide surgical excision. After receiving treatment, the patient showed no evidence of disease for the next 9 years. The study was approved by the Ethics Committee of Mie University, Tsu City, Japan. Written informed consent was obtained from the patient.

## Case report

**Figure 1.**
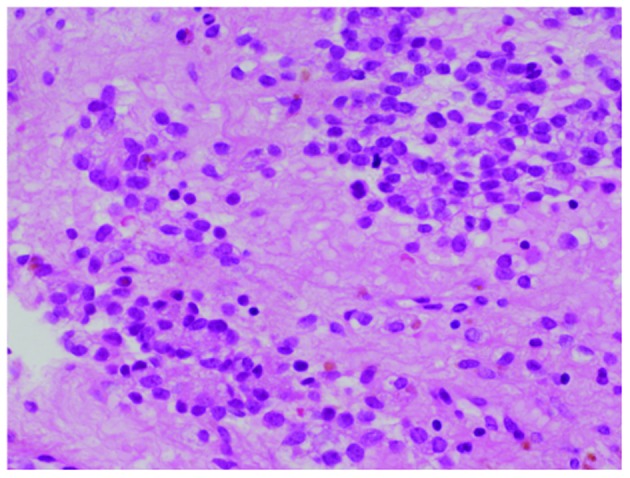
Microscopic findings of the specimen obtained at the initial surgical curettage that was performed at a local hospital when the patient was 20 years old. Hematoxylin and eosin (H&E) staining showed a packed round cell pattern with a striking uniformity. The individual cells had a small round to ovoid nucleus with fine powdery chromatin with few mitotic nucleoli. The cytoplasm was ill-defined and scanty. The histological diagnosis was Ewing’s sarcoma (H&E staining; magnification, ×400).

A 38-year-old male presented with pain in his right thigh. The patient had a past history of treatment for a bone tumor of the right proximal femur. At 20 years of age, the patient had experienced right thigh pain and consulted a doctor at the local hospital. The patient was noted to have a bone tumor of the right femoral bone without distant metastasis and underwent surgical curettage of the tumor. The specimen revealed a malignant small round cell neoplasm with regular nuclear contours, finely dispersed chromatin and scanty cytoplasm without prominent nucleoli (Fig. 1). The patient received one cycle of postoperative chemotherapy that consisted of vincristine and cyclophosphamide and local irradiation with a total dose of 40 Gy. Two years after the initial operation (at 22 years of age), the patient developed severe pain in his right thigh after braking hard while driving. The radiographs taken at the time showed a fracture of the femur where the bone tumor had been located. Open reduction and internal fixation (ORIF) using intramedullary nails was performed. One year after the ORIF, the patient stubbed his toe and could not walk due to severe right thigh pain. Radiographs showed an undisplaced femoral re-fracture. He was conservatively treated and subsequent roentgenograms demonstrated bone union. His subsequent postoperative course had been uneventful for 15 years.

**Figure 2.**
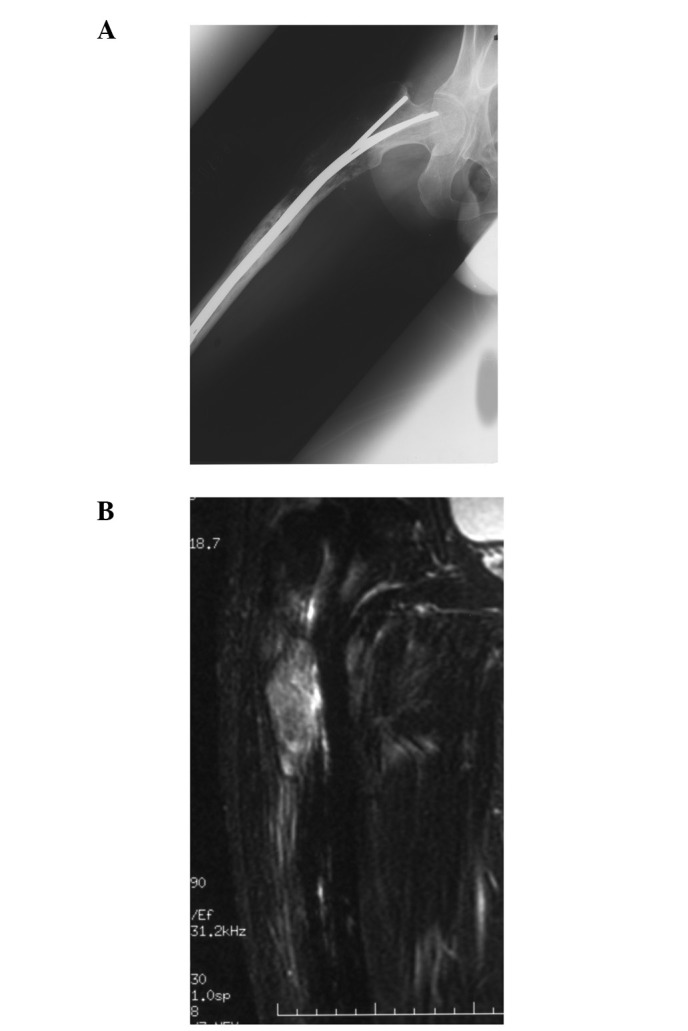
(A) An antero-posterior radiograph showed a lytic lesion from the trochanteric area to the proximal diaphysis. Intramedullary nails had been inserted as the patient had developed a fracture when he was 22 years old. (B) Magnetic resonance imaging showed an intramedullary lesion with an extraskeletal mass.

**Figure 3.**
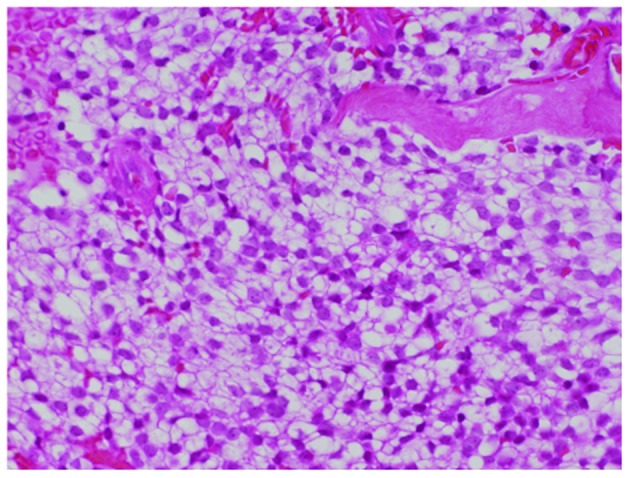
Microscopic findings of the specimen obtained at the open biopsy that was performed at our hospital when the patient was 38 years old. Hematoxylin and eosin (H&E) staining of the recurrent tumor revealed a malignant small round cell tumor similar to the specimen which was obtained at his first operation performed at 20 years of age. The histological diagnosis was recurrent Ewing’s sarcoma family of tumors, based on microscopic, immunohistochemical and histochemical findings, although the molecular biological investigation did not show the presence of any characteristic fusion genes (H&E staining; magnification, ×400).

At 38 years of age, the patient was referred with right thigh pain that had persisted for several months. Radiographs showed a lytic lesion from the trochanteric area to the proximal diaphysis (Fig. 2A). Magnetic resonance images showed an intramedullary lesion with an extraskeletal mass (Fig. 2B) and further metastatic work-up was negative. As local recurrence was suspected, an open biopsy from the intertrochanteric lesion was performed. Microscopic findings revealed a malignant small round cell neoplasm with regular nuclear contours and finely dispersed chromatin without prominent nucleoli (Fig. 3). The microscopic findings were similar to those obtained at first surgery, performed at 20 years of age. Immunohistochemical and histochemical staining showed that the neoplasm was positive for glycogen (PAS), neuron-specific enolase (NSE), MIC-2 and S-100 protein, and negative for α-SMA, CD34, CD56, chromogranin A, neurofilament and vimentin. The MIB-1 index was <10%. Although molecular biological investigations did not reveal the presence of any characteristic fusion genes, including EWS-FLI1, EWS-ERG, EWS-FEV, EWS-ETV1 and EWS-E1AF, a final diagnosis of a local recurrence of ESFT was made. Based on the diagnosis of ESFT, the patient underwent chemotherapy consisting of vincristine, doxorubicin, cyclophosphamide and ifosfamide (12) as well as wide resection of the tumor, combined with reconstruction using a prosthesis. The microscopic findings of the specimen from the widely resected proximal femur revealed a minimum response to the preoperative chemotherapy (necrotic rate <10%). The patient has been disease-free for the past 9 years following this surgery.

## Discussion

ESFT is the second most common primary malignant bone tumor in children and adolescents. Recently, the treatment outcome of ESFT has been improved significantly with the use of multimodal therapy. However, 30–40% of patients with localized disease and 80% of those with metastatic disease succumb due to disease progression.

The present case is unusual for a number of reasons. Firstly, the patient received inadequate initial treatment at the local hospital which consisted of tumor curettage, chemotherapy with an insufficient dose intensity and low-dose radiation therapy. In spite of such inadequate initial treatment, the patient had been disease-free for the subsequent 18 years. The known prognostic factors for ESFT are tumor size or volume, serum LDH levels, axial localization and age (>15 years). Under treatment, a poor histological response to preoperative chemotherapy and incomplete or no surgery for local therapy are adverse prognostic factors (12). One possible reason for the patient being disease-free for the 18 years following the initial treatment is that the radiation therapy may have been extremely effective for this ESFT.

Secondly, the local recurrence occurred 18 years after initial treatment. Local recurrence of malignant bone tumors commonly occurs within the first 1–2 years (14,15) and usually occurs within 3 years of the initial treatment (16,17). Since ESFT is a highly malignant aggressive tumor, local recurrence over fifteen years later is extremely rare. Bacci *et al* (18) reported that 187 of 215 patients (87.0%) relapsed within the first 5 years after starting treatment, while 28 (13.0%) relapsed after more than 5 years. In an analysis of 402 patients followed up for a median of 17.7 years, the longest time from definitive surgery to local recurrence was 7.0 years (18). Donati *et al* (19) reported the longest time until recurrence to be 3.3 years in an analysis of 56 cases. Gasparini *et al* (20) described the long-term outcome in 121 patients with monostotic ESFT treated with combined modality therapy. The mean follow-up time in their study was 12 years, with one patient developing local recurrence at 14 years. No other cases with local recurrence of more than 15 years after the initial treatment were found, except for one case who relapsed 17 years after diagnosis (21).

Thirdly, the microscopic findings of the specimen from the widely resected proximal femur at the most recent surgery revealed only a minimum response to the preoperative chemo-therapy. Despite this, the patient has been disease-free for the past 9 years. Lin *et al* reported that the histological response to preoperative chemotherapy is a strong predictor of local recurrence. Patients who have a poor response (<90% necrosis) have a 50% risk of local recurrence at 5 years (22). Patients with bone or bone marrow metastases and patients with recurrent disease still fare poorly, with 5-year survival rates of 20% (10). This result suggests that the ESFT of our patient had nonmetastatic and non-aggressive biological behavior.

Finally, although the microscopic, immunohistochemical and histochemical findings were typical of ESFT, the molecular biological investigation did not reveal any of the characteristic fusion genes, including EWS-FLI1, EWS-ERG, EWS-FEV, EWS-ETV1 or EWS-E1AF. The diagnosis of the tumor was made using several modalities, such as light microscopy and immunohistochemistry, and was confirmed by several pathologists with specialization in musculoskeletal tumors. The current case, with its unusual clinical course, may involve an unknown fusion gene.

In consideration of both the good clinical course unusual for ESFT and the absence of any of the previously reported fusion genes, the present case may be a rare subtype of ESFT with an unknown chromosomal translocation and relatively non-aggressive biological behavior. Further genetic investigation is therefore required for this patient.
